# Risk Factors for Relapse in Acute Bacterial Prostatitis: the Impact of Antibiotic Regimens

**DOI:** 10.1128/Spectrum.00534-21

**Published:** 2021-09-29

**Authors:** Ester Marquez-Algaba, Carles Pigrau, Pau Bosch-Nicolau, Belen Viñado, Judit Serra-Pladevall, Benito Almirante, Joaquín Burgos

**Affiliations:** a Department of Infectious Diseases, Vall d’Hebron Institut de Recerca (VHIR), Universitat Autonoma de Barcelona, Hospital Universitari Vall d’Hebron, Barcelona, Spain; b Department of Microbiology, Hospital Universitari Vall d’Hebron, Vall d’Hebron Barcelona Hospital Campus, Barcelona, Spain; c Spanish Network for Research in Infectious Diseases (REIPI RD16/0016/0003), Seville, Spain; Riverside University Health System Medical Center, Loma Linda University

**Keywords:** acute bacterial prostatitis, antibiotic resistance, beta-lactams, co-trimoxazole, quinolones, relapse

## Abstract

The aim of the study was to analyze the risk factors for relapse in patients with acute bacterial prostatitis (ABP), focusing on the impact of different antibiotic regimens. We conducted an observational study of all patients diagnosed with ABP (irritative and/or obstructive urinary symptoms, temperature of >37.8°C, and the presence of bacteriuria in urine culture, in the absence of data suggesting pyelonephritis) from January 2017 to December 2018. The main outcome was relapse. We performed a multivariate analysis to identify the risk factors associated with relapse. A propensity score with inverse weighting was applied to attenuate antibiotic selection bias. We included 410 patients. The mean age was 68 years; 28.8% had diabetes mellitus, and 61.1% benign prostatic hyperplasia. The most common isolated bacteria were Escherichia coli (62.4%) and Klebsiella spp. (10%). The overall resistance rate was 39.5% to quinolones. The mortality rate was 1.2%, and the relapse rate was 6.3%. The only independent risk factor for relapse was inadequate antibiotic therapy (odds ratio [OR] 12.3; 95% confidence interval [95% CI], 3.5 to 43.1). When the antibiotic was modified according to the susceptibility pattern, the rates of relapse were 1.8% in those treated with ciprofloxacin, 3.6% with intravenous beta-lactam, 9.3% with co-trimoxazole, and 9.8% with oral (p.o.) beta-lactam (*P* = 0.03). Treatment with oral beta-lactam (OR, 5.3; 95% CI, 1.2 to 23.3) and co-trimoxazole (OR, 4.9; 95% CI, 1.1 to 23.2) were associated with a risk of relapse. In this large real-life observational study, a significantly higher relapse rate was observed when antibiotic treatment was inadequate. When the antibiotic was tailored, quinolones and intravenous beta-lactams had a lower relapse rate than co-trimoxazole and oral beta-lactams.

**IMPORTANCE** In the manuscript, we report a large series of acute bacterial prostatitis cases and describe data about the etiology, antibiotic resistance rate, and outcome, specially focused on the risk factors for relapse. We found high rates of resistance to the most frequently used antibiotics and a high relapse rate in patients whose treatment was not adjusted according to their microbiological susceptibility. We did not observe differences, though, in mortality or relapse according to appropriate or inappropriate empirical treatment. What is new in this article is the different relapse rates observed depending upon the definitive adequate antibiotic used. Quinolones and intravenous (i.v.) beta-lactam have lower rates of relapse (1.8% and 3.6%, respectively) compared to co-trimoxazole and oral (p.o.) beta-lactam (3.3% and 9.8%, respectively). Clinicians should carefully choose an adequate antibiotic for definitive ABP treatment depending on the results of microbiological isolation, using quinolones as the first option. Whenever quinolones cannot be administered, i.v. beta-lactams seem to be the second-best option.

## INTRODUCTION

Acute bacterial prostatitis (ABP) is a common illness and a frequent cause of primary care consultation, hospital admission, and antimicrobial consumption, and it substantially impairs quality of life, especially when it is associated with recurrences (1, 2). Despite its frequency and importance, data specifically focused on this illness are scarce. Most studies regarding febrile urinary tract infections (fUTIs) have been performed in women. However, it is uncertain if the etiology observed and management used in UTIs in the female population are generalizable to those occurring with ABP. Furthermore, the few studies on fUTIs in men include heterogeneous pathology as ABP, acute pyelonephritis, and infection associated with urinary catheters, which may be managed differently (3, 4). The special characteristics of prostate tissue and its distinctive antibiotic penetration conditions mean that management data for other complicated UTIs may not be extrapolated to ABP. Thus, a treatment of insufficient duration and particularly the use of an antibiotic with poor prostatic penetration could lead to an ineffective treatment. Relapse and the progression to chronic bacterial prostatitis (CBP) are nowadays one of the main concerns in the treatment of ABP (5). Currently, the most frequently found pathogen in ABP is Escherichia coli, and a 2 to 4 week course of antibiotics is the recommended treatment (4–7). The properties of fluoroquinolones, with experienced use as well as good prostate penetration, have made them the agents of choice in the management of ABP (5, 6). Nevertheless, the increasing antibiotic resistance of uropathogens, with rates of resistance to quinolones higher than 30%, and the recognition of new adverse effects hinder their use and make it necessary to explore other options (2, 8, 9).

The aim of this study was to explore the clinical outcomes in terms of relapse in patients with ABP, focusing on the impact of different antibiotic regimens. We also aimed to analyze the current etiology and antimicrobial resistance patterns of ABP.

## RESULTS

### Sociodemographic variables, underlying diseases, and clinical presentation.

During the 2-year study period, 863 patients were coded with acute prostatitis. From these patients, just 410 met our definition of ABP and were included in the study.

The mean age of patients was 68 ± 23.9 years; 28.8% (118/410) had diabetes mellitus and 67.6% (277/410) urologic pathology (61.1% [250/410] benign prostatic hyperplasia [BPH], 12% [49/410] urinary incontinence, and 3.9% [16/410] urethral stricture). In all, 16.9% (69/410) of patients had a history of prior urinary manipulation, and 33.3% (133/399) of them had received antibiotic treatment during the previous 3 months for other reasons. On the basis of infection acquisition, 311 (75.9%) of the patients had community-acquired ABP (CA-ABP) and 99 (24.1%) health care-associated ABD (HCA-ABP).

### Etiology of acute prostatitis and antibiotic susceptibility.

A urine culture was obtained from all patients; 41 of 410 (10%) were negative, and in these cases, 83% of the patients had received antibiotics prior to collection of the sample. Less than 1% (4/410) were contaminated. Blood cultures were performed in 363 of the 410 patients and were positive in 82 cases (20% of all patients).

The most common microorganism isolated for CA-ABP was E. coli (67.5%), followed by Klebsiella spp. (9.2%). For HCA-ABP, E. coli was isolated in 43.5% of patients, followed by Pseudomonas aeruginosa in 18.5%.

Regarding antibiotic susceptibility, the overall rates of resistance of the isolates to quinolones were 33.6% for CA-ABP and 55.3% for HCA-APB (*P* < 0.001). Higher rates of resistance to beta-lactams and co-trimoxazole were also observed in HCA-ABP and in patients with previous use of antibiotics (data not shown).

Data on the causative agents of ABP and the specific antibiotic susceptibility of each microorganism isolated are shown in Table 1.

**TABLE 1:** Etiology and antibiotic resistance rates in CA-ABP and HCA-ABP

Antibiotic^*a*^	Resistance isolates (*n*/*N* [%])^*b*^ for:		
	Escherichia coli	*Klebisella* spp.	Proteus spp.
Total CA-ABP^*c*^	211/313 (67.4)	29/313 (9.3)	11/313 (3.5)
Amoxicillin-clavulanic acid	78/211 (37.1)	4/29 (13.8)	4/11 (36.4)
Cefuroxime	47/211 (22.3)	3/29 (10.3)	2/11 (18.2)
Cefotaxime	30/211 (14.3)	3/29 (10.3)	1/11 (9.1)
Ciprofloxacin	79/211 (37.4)	3/29 (10.3)	4/11 (36.4)
Co-trimoxazole	59/211 (28)	1/29 (3.4)	5/11 (45.5)
Ertapenem	0	0	0
Fosfomycin	2/203 (0.9)	5/29 (17.2)	1/11 (9.1)
ESBL resistance mechanism	27/211 (12.8)	2/29 (6.9)	0
Total HCA-ABP^*d*^	47/109 (43.1)	20/109 (18.3)	14/109 (12.8)
Amoxicillin-clavulanic acid	27/47 (57.4)	20/20 (100)	4/14 (28.6)
Cefuroxime	22/47 (46.8)	20/20 (100)	3/14 (21.4)
Cefotaxime/ceftazidime	16/47 (34)	7/20 (35)	3/14 (21.4)
Ciprofloxacin	34/47 (72.3)	8/20 (40)	4/14 (28.6)
Co-trimoxazole	20/47 (42.6)	20/20 (100)	2/14 (14.3)
Ertapenem	0	20/20 (100)	0
Meropenem	0	3/20 (15)	0
Fosfomycin	1/47 (2.1)	20/20 (100)	3/14 (21.4)
ESBL resistance mechanism	15/47 (31.9)	0	2/14 (14.3)

a CA-ABP, community-acquired acute bacterial prostatitis; HCA-ABP, health care-acquired acute bacterial prostatitis; ESBL, extended-spectrum beta-lactamase-producing *Enterobacteriaceae*, defined as third-generation cephalosporin resistance.

b Twelve patients had a urine culture positive for two microorganisms, so the total number of isolates is 422.

c Other etiology of CA-ABP: *Citrobacter* spp., 2.2%; Enterococcus faecalis, 1.9%; other microorganisms, 3.2%; and negative or contaminated urine, 12.8%.

d Other etiology of HCA-ABP: *Enterococcus* spp., 8.3%; Enterobacter spp., 4.6%; other microorganisms, 8.2%; and negative or contaminated urine, 4.6%.

### Antimicrobial therapy.

The most frequently administered empirical antibiotic was ciprofloxacin (188/408 [46.1%]), followed by cefuroxime (91/408 [22.3%]) and third-generation cephalosporins (43/408 [10.3%]). Empirical treatment was considered inadequate in 25.6% of cases due to a resistant microorganism.

Once the microbiological results were obtained, the treatment was assessed as suitable or was modified according to the susceptibility pattern in 347 cases. In 63 cases, the treatment was not tailored. Of these patients, 71.4% had a negative or contaminated urine culture, 15.8% were lost to follow-up, and 12.6% received an incorrect treatment.

Once modified according to susceptibility, the most frequent treatment was ciprofloxacin 500 mg twice a day (b.i.d.) (190/348 [54.6%]), followed by oral (p.o.) beta-lactams (cefixime 400 mg once a day [q.d.], cefuroxime 500 mg three times a day [t.i.d.], or amoxicillin-clavulanic acid 875/125 mg t.i.d.) (58/348 [16.7%]), intravenous (i.v.) beta-lactams (ertapenem 1 g q.d. or ceftriaxone 2 g q.d.) (57/348 [16.4%]), and co-trimoxazole 160/800 mg b.i.d. (43/348 [12.4%]). The median duration of treatment was 21 days (interquartile range [IQR], 18 to 21).

### Clinical outcome and relapse.

The median hospital stay was 4 days (1–7). No patient presenting with septic shock required vasoactive drugs. Only two patients (0.5%) required intensive care unit (ICU) admission. Five elderly patients (1.2%) with many comorbidities died. Three of them did not receive adequate empirical treatment at admission, but this was adjusted within the first 48 h after admission. Only one death was directly attributable to sepsis due to ABP.

### Risk factors for relapse and impact of antibiotic therapy.

Overall recurrence was observed in 39 of 366 (14.3%) patients for whom data were recorded. Relapse occurred in 23 patients (6.3%), and 16 (4.4%) were diagnosed with reinfection. In the multivariate analysis of all patients, after adjustment for different factors, the only independent risk factor for relapse was inadequate definitive antibiotic treatment (odds ratio [OR], 12.3; 95% confidence interval [95% CI], 3.5 to 43.1; *P* < 0.001). Although not statistically significant, BPH showed a trend as a risk factor for relapse (OR, 2.9; 95% CI, 0.9 to 9.5; *P* = 0.07). The sensitivity propensity score analysis confirmed the risk of relapse with inadequate antibiotic treatment (OR, 16.67; 95% CI, 4.54 to 50; *P* < 0.001). The results are shown in Table 2.

**TABLE 2:** Risk factors for relapse in all patients

Risk factor		Univariate analysis^*a*^		Multivariate analysis	
		Relapses (*n*/*N* [%])	*P* value	Relapse OR (95% CI)	*P* value
Adequate empirical treatment^*b*^	No	12/97 (12.4)	<0.001	2.4 (0.9–6.2)	0.064
	Yes	11/264 (4.2)			
Adequate definitive treatment^*b*^	No	6/16 (37.5)	<0.001	12.3 (3.5–43.1)	<0.001
	Yes	16/339 (4.7)			
Prostatic hypertrophy	Yes	19/228 (8.3)	0.026	2.9 (0.9–9.5)	0.071
	No	4/137 (2.9)			
Diabetes mellitus	Yes	10/105 (9.5)	0.087	1.8 (0.7–4.6)	0.245
	No	13/261 (5)			
Short antibiotic treatment^*c*^	Yes	3/76 (3.9)	0.240	1.3 (0.4–5)	0.628
	No	20/282 (7.1)			

a Variables not included in the multivariate model were as follows (% relapse versus nonrelapse, respectively): age > 65 years (7% versus 5%, *P* = 0.296), immunosuppression (0% versus 6.7%, *P* = 0.200), previous antibiotic exposure (8.1% versus 5.4%, *P* = 0.225), intravenous treatment (4.9% versus 6.6%, *P* = 0.446), positive blood culture (3.7% versus 6.8%, *P* = 0.483), and urinary tract manipulation (7.6% versus 6%, *P* = 0.405).

b We considered antibiotic treatment adequate if it was tailored according to the urine culture result and antibiotic susceptibility testing (considered sensitive according to EUCAST 2019). Cases in which the antibiotic could not be adjusted by negative urine culture were excluded from the analysis.

c Duration of short treatment, ≤14 days.

In the subgroup of patients with adequate definitive antibiotic treatment, the rates of relapse were 1.8% in patients treated with ciprofloxacin, 3.6% with i.v. beta-lactam, 9.3% with co-trimoxazole, and 9.8% with p.o. beta-lactam. The results are shown in Table 3.

**TABLE 3:** Risk factors for relapse in patients with adequate treatment

Risk factor	Univariate analysis		Multivariate analysis	
	Relapses (*n*/*N* [%])	*P* value	Relapse OR (95% CI)	*P* value
Diabetes mellitus				
Yes	7/97 (7.2)	0.139	1.2 (0.4–3.9)	0.731
No	9/242 (3.7)			
Prostatic hypertrophy				
Yes	14/211 (6.6)	0.026	3.2 (0.7–15.2)	0.130
No	2/127 (1.7)			
Adequate definitive antibiotic treatment^*a*^				
Quinolone	3/171 (1.8)	0.030	1	
Beta-lactam (i.v.)	2/55 (3.6)		1.7 (0.3–10.7)	0.568
Co-trimoxazole	4/43 (9.3)		4.9 (1.1–23.2)	0.044
Beta-lactam (p.o.)	5/51 (9.8)		5.3 (1.2–23.3)	0.029
Short antibiotic treatment^*b*^				
Yes	2/75 (2.7)	0.260		
No	14/258 (5.4)			
Adequate empirical treatment^*c*^				
Yes	9/255 (3.5)	0.069		
No	7/79 (8.9)			

a We considered the antibiotic treatment to be adequate if it was tailored according to the urine culture result and antibiotic susceptibility testing (considered sensitive according to EUCAST 2019). Cases in which the antibiotic could not be adjusted by negative urine culture were considered inadequate antibiotic treatment.

b Treatment duration, ≤14 days.

c This variable was not included in the multivariate analysis due to high colinearity.

In comparison with quinolone treatments, the multivariate analysis found that treatment with p.o. beta-lactam (OR, 5.3; 95% CI, 1.2 to 23.3; *P* = 0.029) or co-trimoxazole (OR, 4.9; 95% CI, 1.1 to 23.2; *P* = 0.044) were independent predictors of relapse. The propensity score model confirmed this finding. The data are shown in Table 4.

**TABLE 4:** Risk for relapse in patients with adequate treatment according to antibiotic regimen

Adequate definitive antibiotic treatment^*a*^	Propensity score	
	Relapse OR (95% CI)	*P* value
Quinolone	1	
Beta-lactam (i.v.)	1.64 (0.24–11.27)	0.667
Co-trimoxazole	3.31 (0.61–18.06)	0.166
Beta-lactam (p.o.)	5.47 (1.12–26.66)	0.036

a We considered antibiotic treatment to be adequate if it was tailored according to the urine culture result and antibiotic susceptibility testing (considered sensitive according to EUCAST 2019 [26]).

## DISCUSSION

In this large observational study, the main finding is the impact of the antibiotic regimen on the risk of relapse, in this era of high rates of antibiotic resistance.

One of the main concerns regarding ABP is the rate of relapse of the disease. In our study, we observed a relapse rate of 6.3%, similar to the 5% to 10% published in previous studies (10, 11). Diabetes mellitus, BPH, incontinence, and a history of prior UTI have been proposed as risk factors for relapse or progression to chronic prostatitis (10, 11). Prostate hypertrophy conditions incomplete urination and makes patients more susceptible to infection, and it appears to produce a lower cure rate with an increased risk of relapse. Although not statistically significant, we also observed a tendency toward a higher risk of relapse in such patients.

Choosing an appropriate empirical treatment for ABP can be challenging given the high rates of resistance. According to the IDSA guidelines, local rates of resistance to any antibiotic should not exceed 10% to consider an antibiotic as appropriate for UTI empirical therapy (12), and for this reason, some authors suggest not giving quinolones (13). However, these recommendations are based on expert opinion with limited evidence to support them. In our unit, we did not observe differences in clinical outcomes and risk of relapse associated with appropriate or inappropriate empirical treatment. Our data are supported by two multinational studies that showed that inappropriate empirical antibiotic therapy was neither associated with treatment failure nor mortality (3, 14). Different explanations can be provided; it is probable that the antibiotics used may retain some activity *in vivo* against isolates, despite the phenotypic resistance found *in vitro* (15, 16). Also, severe clinical presentation of ABP is rare. Rates of septic shock and mortality in ABP are lower than 3%, according to different studies (8, 14, 17, 18). In nonsevere illness, the impact of initiating an appropriate empirical antibiotic should be less than that of ensuring an adequate definitive antibiotic. Thus, and to avoid overuse of third-generation cephalosporins or carbapenems, the use of antibiotics for which there is a relatively high rate of resistance, such as second-generation cephalosporins or quinolones, could be considered empirically, at least in outpatient treatment or in nonsevere hospitalized patients. In patients with ABP who present with severe disease, third-generation cephalosporins or carbapenems seem a more appropriate therapy until microorganism results are available.

Moreover, the most important risk factor for relapse was the nonadjustment of definitive therapy. Patients whose treatment was not tailored according to their antimicrobial susceptibility had a significantly increased risk of relapse, 12 times higher than in patients for whom the therapy was adjusted. Thus, the most important approach to the management of ABP is not just applying empirical treatment, but modifying it according to microbiological isolation results. A greater effort should be made to collect adequate urinary samples before starting empirical antibiotic treatment, as well as to ensure the correct follow-up of patients with ABP.

The optimal duration of antibiotic therapy in ABP is still under discussion. Clinical guidelines recommend a 2 to 4 week course of treatment for ABP, although strong evidence is lacking (4–7). One study linked short-duration treatment to an increased risk of developing chronic prostatitis (10). However, other studies suggest that a 2-week course of antibiotics is not associated with increased rates of relapse (5, 11). We agree that the duration of treatment should be decided according to the severity of the disease and treatment response (19). In our hospital, we propose 2 weeks of antibiotic treatment in those patients with a rapid clinical response and without factors associated with a poor prognosis and extending the treatment to 3 weeks in the rest of the patients. Longer antibiotic treatment is recommended in cases of prostate abscesses or suspected chronic bacterial prostatitis.

Furthermore, the most important finding of our study was the different relapse rates observed depending upon the definitive adequate antibiotic used, confirmed by a propensity score analysis. Most guidelines recommend the use of quinolone as a definitive antimicrobial therapy and the use of co-trimoxazole as an alternative whenever quinolone cannot be administered (5, 20, 21). These recommendations are based on clinical experience and the pharmacokinetic/pharmacodynamic (PK/PD) properties of drugs in prostate tissue (5, 22). Our study showed that ABP treated with quinolones has a lower rate of relapse, close to 1%. Unexpectedly, we found that treatment with co-trimoxazole was associated with a higher risk of relapse, around 10%, and comparable to p.o. beta-lactam treatment. A possible explanation is that, although trimethoprim levels in prostatic fluid may exceed the levels in plasma, the prostatic concentration of sulfamethoxazole is much lower, raising doubts that it synergizes with trimethoprim (5, 23, 24). In fact, a study performed in the United States in more than 33,000 older men with a UTI diagnosis described a higher relapse rate in patients treated with co-trimoxazole compared to quinolones (11). Until more data about co-trimoxazole is available, we suggest administering higher doses (160/800 mg t.i.d.), at least in overweight patients. Regarding beta-lactams, it is known that they have a low oral bioavailability and poor penetration into prostate tissue. Thus, if administered orally, adequate concentrations in the prostate may not be achieved, and this confers a greater risk of relapse, especially in strains with MIC values close to the resistance breakpoint (5). In summary, clinicians should carefully choose an adequate antibiotic for definitive ABP treatment, dependent on the results of microbiological isolation, using quinolones as the first option. Whenever quinolone cannot be administered, i.v. beta-lactams seem to be the second-best option.

The major strength of our study is that we present a large series of adult men with ABP, with a correlation between antibiotic treatment and clinical outcome. Studies focused specifically on the clinical course of ABP are scarce, and this work provides new information. However, our study has some limitations, mainly because it derives from real-life analysis. First, this is a single-hospital-based study, and the data, especially regarding antimicrobial susceptibility, may not be extrapolable to other areas. Furthermore, there is a current lack of agreement regarding guidelines for the diagnosis and treatment of acute bacterial prostatitis, making extrapolation of the data more difficult. Second, there may be a treatment indication bias, since the reason why physicians chose certain regimens was not controlled. Ideally, a randomized trial should be performed to compare empirical regimens; however, our propensity score approach allows attenuation of the treatment indication bias. Finally, as the study was retrospectively designed, some data from the primary care database could have been missed, and the clinical efficacy of the different antibiotics may be biased.

In conclusion, there are high rates of resistance to the most frequently used antibiotics to treat ABP. The relapse rate is high in patients whose treatment is not modified according to the microbiological susceptibility. When modified, quinolone and i.v. beta-lactam treatment have higher cure rates than co-trimoxazole and p.o. beta-lactams. Properly designed clinical trials are required in order to assess the efficacy of different antimicrobials, including other alternatives poorly explored in ABP, such as fosfomycin.

## MATERIALS AND METHODS

### Study population and setting.

We conducted a retrospective observational study, collecting data from all consecutive adult patients (aged >16 years) diagnosed with ABP at the emergency department or during hospitalization from January 2017 to December 2018 at Vall d’Hebron University Hospital (Barcelona, Spain), a 1,000-bed center. We selected patients according to hospital diagnosis codes (the included codes were 601.0, N41.0, N41.2, N41.3, N41.8, and N41.9 of the *International Classification of Diseases*, ninth and tenth revisions [ICD-9 and ICD-10]) (25). The medical records of selected patients were reviewed, including only those who met the definition of ABP in the study. The study protocol was approved by the institutional ethics board at Hospital Universitari Vall d’Hebron, Barcelona (PR[AG]626/2020).

### Study variables and data collection.

For data collection, we accessed medical and nursing records from hospital and primary health centers. Demographic data, comorbidities (renal impairment, diabetes mellitus, immunosuppression, etc.), urological diseases (benign prostatic hyperplasia, urethral stricture, or urinary incontinence), health care-associated factors, clinical presentation and relapse, previous antibiotic exposure, microbiological data, antimicrobial therapy, and outcomes (mortality, clinical cure, relapse, and reinfection) were recorded from each episode.

### Outcome measures and definitions.

The primary outcome assessed was the risk of relapse of ABP within the next 3 months of follow-up. The secondary outcomes were analysis of the impact of the antibiotic regimen on the risk of relapse and description of the etiology and resistance patterns of the microorganisms causing ABP.

ABP was defined when the following criteria were fulfilled: (i) abrupt presentation of voiding symptoms (irritative and/or obstructive), (ii) temperature of >37.8°C, (iii) the presence of bacteriuria in a clean-catch midstream urine specimen without prostate massage, and (iv) the absence of data suggestive of pyelonephritis (costovertebral angle tenderness).

We excluded cases of fever presenting within 24 h of urinary tract manipulation. We included permanent indwelling catheter-related infections only if prostatitis was confirmed by digital rectal examination or compatible radiology. Cases suggestive of chronic bacterial prostatitis (CBP) were also excluded, defining CBP as (i) bacterial prostatitis of long duration (symptoms lasting more than 4 weeks) or (ii) two or more episodes of acute prostatitis caused by the same organism during the last 12 months.

For the purpose of this study, relapse was defined as a recurrence with the same microorganism and antibiotic susceptibility pattern during the next 3 months of follow-up (10). Reinfection was defined as a recurrence caused by a different microorganism in a period of 3 months.

The antibiotic treatment was considered adequate if it was suitable or modified according to the urine culture result and antibiotic susceptibility pattern (considered susceptible according to the European Committee for Antimicrobial Susceptibility Testing [EUCAST] 2019 [24]). We differenced between empirical and definitive antibiotic treatment. Cases in which the antibiotic could not be tailored due to contaminated or negative urine culture were excluded from the analysis.

### Microbiological procedures.

A positive urine culture was defined as the isolation of a uropathogen with a bacterial count of >10^4^ CFU/ml. Urine cultures positive for 2 microorganisms were taken into account when both of them were common uropathogens, with a growth of >10^4^ CFU/ml (1). Any counts below this with ≥2 organisms present were considered contamination. The antibiotic susceptibility testing was performed using the automated Vitek 2 system (bioMérieux, France). The MIC values for penicillins (ampicillin, amoxicillin-clavulanic acid, and piperacillin-tazobactam), cephalosporins (cefuroxime and cefotaxime), carbapenems (ertapenem, imipenem, and meropenem), amikacin, ciprofloxacin, trimethoprim-sulfamethoxazole, and fosfomycin were interpreted retrospectively according to criteria established by the EUCAST 2019 version 9.0 guidelines (26). When the susceptibility was defined by the laboratory as intermediate or an area of technical uncertainty (ATU), the isolate was considered resistant to this antibiotic.

### Statistical analysis.

The epidemiological and clinical characteristics and outcome variables of all individuals with ABP were included in the descriptive analysis and compared using the Pearson χ^2^ test or the Fisher exact test for categorical variables and the Student *t* test or Mann-Whitney U test for continuous variables. The descriptive results are presented as median and interquartile range (IQR) or means ± standard deviation (SD) and as frequencies and percentages for categorical variables.

To identify the risk factors for relapse, a logistic regression analysis was performed. Variables showing statistically significant differences associated with relapse in the univariate analysis and those clinically relevant were included in the multivariate models, in a sequential manner. Variables with high colinearity in the bivariate correlation were excluded from analysis. For the purpose of the study, the multivariate analysis was performed (i) on the whole population and (ii) only on patients with adequate definitive antibiotic treatment.

Finally, to assess the impact of different antibiotic regimens and to attenuate selection bias, we applied the propensity score using the inverse probability of the treatment-weighting method. Propensity score weights were determined using a multinomial regression model.

Odds ratios (ORs) with 95% confidence intervals are reported. All statistical tests were two-tailed, and the threshold of statistical significance was *P* < 0.05. Statistical analyses were performed using SPSS Statistics version 23.0 software.
